# Resection of the vaginal vault for vaginal recurrence of cervical cancer after hysterectomy and brachytherapy

**DOI:** 10.1186/s12957-015-0495-8

**Published:** 2015-04-02

**Authors:** Akiko Abe, Maki Matoda, Sanshiro Okamoto, Eiji Kondo, Kazuyoshi Kato, Kohei Omatsu, Kenji Umayahara, Kuniko Utsugi, Nobuhiro Takeshima

**Affiliations:** Departments of Gynecology, Cancer Institute Hospital, 3-8-31 Ariake, Koto-ku, Tokyo 135-8550 Japan

**Keywords:** Cervical cancer, Vaginal recurrence, Radiotherapy, Vaginal vault resection, Postoperative complications

## Abstract

**Background:**

We describe our experiences with vaginal vault resection for vaginal recurrence of cervical cancer after hysterectomy and radiotherapy. After operative treatment, the rate of vaginal vault recurrence of uterine cervical cancer is reported to be about 5%. There is no consensus regarding the treatment for these cases.

**Methods:**

Between 2004 and 2012, eight patients with vaginal vault recurrence underwent removal of the vaginal wall via laparotomy after hysterectomy and radiotherapy.

**Results:**

The median patient age was 45 years (range 35 to 70 years). The median operation time was 244.5 min (range 172 to 590 min), the median estimated blood loss was 362.5 mL (range 49 to 1,890 mL), and the median duration of hospitalization was 24.5 days (range 11 to 50 days). Two patients had intraoperative complications: a grade 1 bowel injury and a grade 1 bladder injury. The following postoperative complications were observed: one patient had vaginal vault bleeding, three patients developed vesicovaginal fistulae, and one patient had repeated ileus. Two patients needed clean intermittent catheterization. Local control was achieved in five of the eight cases.

**Conclusions:**

Vaginal vault resection is an effective treatment for vaginal recurrence of cervical cancer after hysterectomy and radiotherapy. However, complications of this procedure can be expected to reduce quality of life. Therefore, this operation should be selected with great care.

## Background

Cervical cancer is the fourth most common cancer in women worldwide. In 2012, 528,000 cases of cervical cancer were diagnosed globally and 266,000 deaths were attributed to this disease [1]. In Japan, the incidence of early stage cervical cancer is increasing among younger women [2]. There are many long-term survivors of these cancers, and those who experience locoregional recurrence after initial surgery may require radiotherapy [3]. In most cases, radiotherapy can control vaginal vault recurrence [4,5], but additional relapse is occasionally experienced. If it is feasible, surgical resection is generally considered for such cases. Vaginal vault resection is a technique that is applicable to rare clinical cases. Only a few reports have described the efficacy of laparoscopic or vaginal vaginectomy [6,7]. To the best of our knowledge, this is the first study to report on vault resection performed via laparotomy. In the present study, we aimed to describe the efficacy and feasibility of vaginal vault resection by laparotomy for isolated small vaginal relapse of cervical cancer.

## Methods

Eight patients were evaluated, each of whom experienced isolated vaginal relapse of uterine cervical cancer, and each of whom subsequently underwent vaginal vault resection by laparotomy at Cancer Institute Hospital (Tokyo, Japan) between 2004 and 2012. This research was performed with the approval of the Regional Ethics Committee for Clinical Study of the Cancer Institute Hospital, Tokyo, Japan, and in compliance with the Helsinki Declaration. The medical records of these patients were reviewed retrospectively. Table 1 shows the patients’ characteristics. Four patients were referred to our institute after prior hysterectomy, and four patients had undergone hysterectomy at our hospital. Hysterectomy was performed as simple extrafascial abdominal hysterectomy (four patients), vaginal hysterectomy (one patient), modified hysterectomy (two patients), or radical hysterectomy (one patient). In four patients, hysterectomy was combined with bilateral salpingo-oophorectomy, and in three patients, it was combined with pelvic lymphadenectomy.

**Table 1:** **Clinical features of the patients at the initial operation**

	**Case 1**	**Case 2**	**Case 3**	**Case 4**	**Case 5**	**Case 6**	**Case 7**	**Case 8**
Age	69	35	43	41	33	30	44	49
Initial stage	0	0	0	Ia	Ia	Ia	IIa	IIb
Histology	AS *in situ*	CIS	AIS	SCC	AC	SCC	SCC	AS
Initial operation	TAH	TAH	MRH + BSO + PLA	TAH	RH + BSO + PLA	VTH	MRH + BSO	RH + BSO + PLA
Vaginal margin	Negative	Positive	Negative	NA	Negative	Positive	Positive	NA
Adaptation of radiation treatment	Adjuvant	Recurrence	Recurrence	Adjuvant	Recurrence	Recurrence	Adjuvant	Recurrence
Interval between initial operation and vaginectomy	12 M	13 M	44 M	12 M	9 M	38 M	12 M	10 M

AS, adenosquamous cell carcinoma; CIS, carcinoma *in situ*; AIS, adenocarcinoma *in situ*; SCC, squamous cell carcinoma; AC, adenocarcinoma; TAH, total abdominal hysterectomy; MRH, modified radical hysterectomy; BSO, bilateral salpingo-oophorectomy; PLA, pelvic lymphadenectomy; RH, radical hysterectomy; VTH, vaginal total hysterectomy; NA, not available.

Some patients had been treated with radiation using remote afterloading systems for positive margins of hysterectomy, and other patients had been treated for first vaginal vault recurrence after hysterectomy. After brachytherapy, some patients developed vaginal vault recurrence. Recurrences were treated with vaginal vault resection by laparotomy in eight cases. The intervals between brachytherapy and vaginal vault recurrences varied from 9 to 44 months. The patients were asked to indicate that they understood the nature of the surgical procedure, operative complications, and postoperative complications.

**Figure 1:**
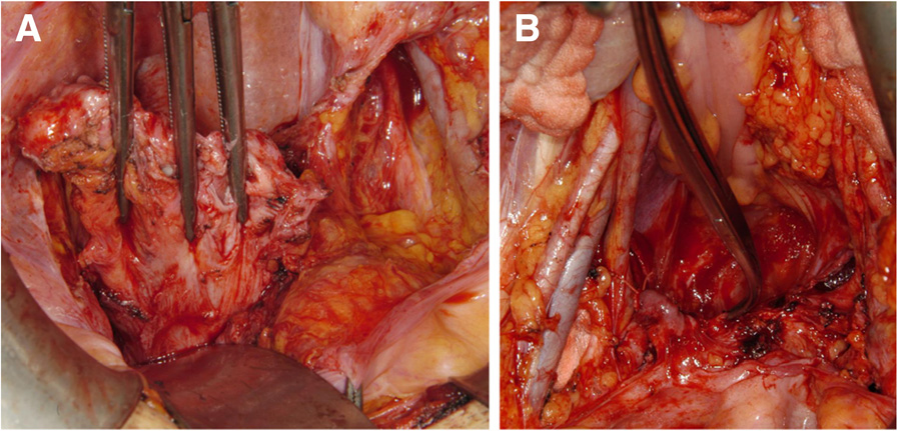
**The vaginal apex identified using Hegar sounds and pulled via the apex (A) and pelvic appearance after vaginectomy (B).**

**Figure 2:**
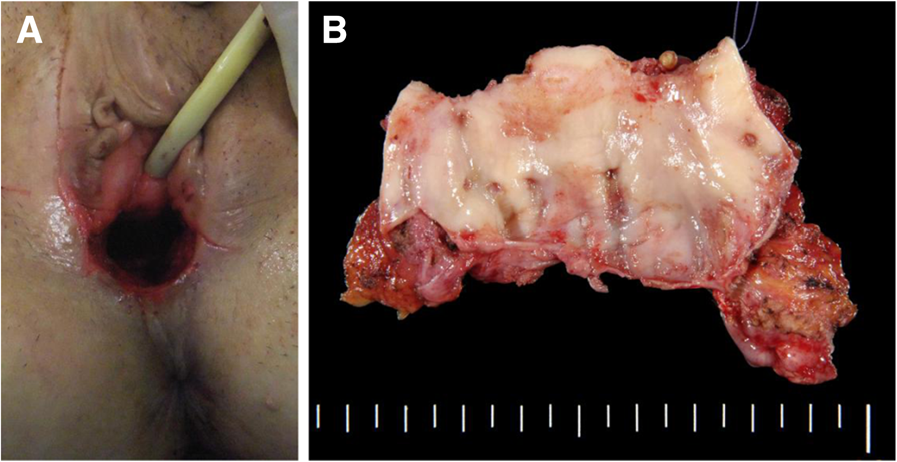
**The vaginal appearance after the operation (A) and the dissected vaginal specimen (B).**

Surgical procedures were performed as follows (Figures 1 and 2). After bringing the patient into the Trendelenburg position, bilateral ureteral catheters and a Foley catheter were inserted under general anesthesia. Laparotomy was then performed, and exploration of the abdomen did not reveal other lesions in any of the cases. Adhesiolysis was performed between the rectum and the scar from the previous surgery. The pararectal and paravesical spaces were developed, and the surface of the levator ani muscle was exposed. Division between the posterior vaginal wall and the rectum was performed close to the muscular layer of the rectum, and this dissection plane was continued to the perineal body. The vaginal apex was identified using Hegar sounds and pulled via the apex. Ventral to the vagina, the plane of excision was placed in line with the limit of the muscular layer of the bladder. Subsequently, we resected the parametrium lateral to the vagina with the intention of preserving the nerve branches running from the inferior hypogastric plexus into the bladder. The vaginal wall was exfoliated via the transvaginal approach, and the vaginal vault was totally removed. Subsequently, the dissected end of the levator ani muscle was closed with sutures. We did not close the perineal opening, which was the dissecting line of the lower edge of the vagina. Finally, the bladder was filled with saline to check for leakage.

## Results

Patient characteristics at the initial operation are summarized in Table 1. The median patient age was 45 years (range 35 to 70 years), the median body mass index was 19.7 (range 16.8 to 24.5), and all patients met the criteria for American Society of Anesthesiologists physical status (PS) classification 1 to 2. Three patients were diagnosed with carcinoma *in situ*: one patient was diagnosed with carcinoma *in situ*, one patient was diagnosed with adenosquamous cell carcinoma *in situ*, and one patient was diagnosed with adenocarcinoma *in situ*. Three patients had stage Ia disease: two had squamous cell carcinoma, and one had adenocarcinoma. Two patients had stage II disease: one had squamous cell carcinoma and one had adenosquamous cell carcinoma. Vaginal surgical margins were positive in three cases, negative in three cases, and unavailable in two cases.

Table 2 presents operative data regarding resection of the vaginal vault and complications. The surgical margins were positive in four cases, negative in three cases, and unclear in one case, as caused by coagulation. The median operation time was 244.5 min (range 172 to 590 min), and the median estimated blood loss was 362.5 mL (range 49 to 1,890 mL). Bilateral ureteral catheters were inserted in seven patients. The Foley catheter was removed after a median of 7 days (range 3 to 20 days). Two patients needed clean intermittent catheterization after removal of the Foley catheter. The median hospital stay was 24.5 days (range 11 to 50 days).

**Table 2:** **Clinical feature of the patients at vaginectomy**

	**Case 1**	**Case 2**	**Case 3**	**Case 4**	**Case 5**	**Case 6**	**Case 7**	**Case 8**
Age	70	36	48	44	35	36	46	54
Operative time (min), for vaginectomy	180	590	219	172	270	316	191	442
Blood loss, mL	250	1,890	49	150	365	490	360	760
Intraoperative complications	Rectal injury G1	None	Bladder injury G1	None	None	None	None	None
Postoperative complications	Cystitis	Vesicovaginal fistula (POD33)	Vesicovaginal fistula (POD40), ileus, nephritis	None	None	Nephritis	Vesicovaginal fistula (POD19), nephritis	Stump bleeding
Self-catheterization	No	No	No	No	+	No	No	+
Margin of resection	Positive	Positive	Unclear	Positive	Negative	Positive	Negative	Negative
Site of recurrence after colpectomy	PLN	Pelvic wall	Vulva	No	No	PAN	No	Vulva PLN
Treatment for recurrence after colpectomy	Radiation	Exenteration	Vulvectomy			Chemotherapy		Vulvectomy + lymphadenectomy
Time to recurrence from colpectomy	10 M	2 M	6 M			22 M		19 M
Final status	AWD	DOD	AWD	NED	NED	AWD	NED	AWD

POD, postoperative day; PLN, pelvic lymph node; PAN, para-aortic lymph node; AWD, alive with disease; DOD, dead of disease; NED, no evidence of disease.

**Figure 3:**
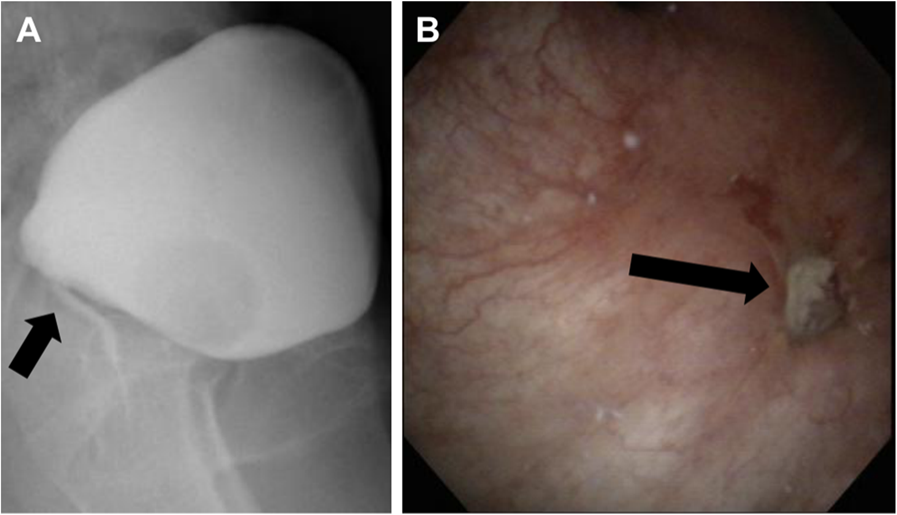
**Urethrocystography showing a vesicovaginal fistula (arrow) (A, B).**

With regard to postoperative complications, one patient experienced vaginal vault bleeding. Another patient experienced repeated ileus. Three patients developed vesicovaginal fistulae (Figure 3). For each of these patients, the Foley catheter was kept in place for 3 months, after which the fistula spontaneously closed. The median follow-up period after the resection of vault failure was 44.5 months (range 22 to 76 months). Regarding posttreatment surveillance, physical examinations were performed, with cytological investigation of the vaginal vault and transvaginal ultrasound (every 2 months for 2 years and every 4 months for another 3 years thereafter). After vaginal vault resection, three patients were disease-free and had good general condition. Five other patients developed recurrence: two developed distant metastasis, two developed locoregional failure, and one developed locoregional and distant metastasis. The median time to recurrence after vaginal resection was 10 months (range 2 to 22 months).

Two patients showed lymph node involvement after treatment for relapse. One of these patients had pelvic lymph node metastasis and was treated with radiotherapy. The other had para-aortic lymph node metastasis and was treated with chemotherapy. At the last follow-up, both patients were alive and well, with no evidence of disease. A patient with locoregional failure showed recurrence in the vagina, and pelvic exenteration and ureteroileostomy were performed. This patient died due to peritoneal dissemination 23 months after vaginal resection. A patient with locoregional and distant failure showed recurrence in the vulva after vaginal vault resection. The tumor was partially resected from the vulva. Furthermore, this patient developed multiple liver metastases 30 months later. With respect to postoperative complications, repeated ileus was managed with some difficulty. After a symptom was improved by fasting, this patient was treated with TS-1, resulting in the shrinkage of the recurrent tumors, and proton beam therapy was performed. Forty-five months after vaginal vault resection, the patient was alive with disease at the last follow-up.

## Discussion

The rate of vaginal vault recurrence of stages I to II uterine cervical cancer after operative treatment has been reported to be 4.9% [8]. No consensus has been reached regarding the selection of multimodal treatment strategies. In general, radiotherapy is effective in terms of local control with vaginal vault recurrence. We treated first vaginal relapse with radiotherapy, and although 95% patients were cured at our hospital, we sometimes observed second vaginal failures after providing radiotherapy for first vaginal relapse.

In a prior study, Berek *et al*. noted that intent-to-cure recurrent cervical carcinoma was the indication for pelvic exenteration after prior pelvic radiotherapy [9]. Although they found pelvic exenteration to be a feasible surgical procedure, it was associated with severe complications. Accordingly, Berek *et al*. described using pelvic exenteration as a last option for surgical therapy.

Panici *et al*. reported that the complications of pelvic exenteration include the development of ileus in 42% of patients (5/12), the requirement of re-laparotomy in 17% of patients (2/12), and the occurrence of leaks after intestinal anastomosis in 37.5% of patients (3/8) [7]. Panici *et al*.’s report further showed that the median operative time was 491.5 min, the estimated blood loss was 2537.5 mL, and the median hospital stay was 65.5 days. Another report has also shown similar results, including major and minor complication rates of 34% and 57%, respectively [10]. Complete resection with exenteration is a significant prognostic factor for local control and survival, but it is also associated with high morbidity.

We have treated first vaginal relapse with radiotherapy and performed surgery for second vaginal failures. In consideration of morbidity, we have used surgery for resection of the vaginal vault, instead of using pelvic exenteration. The indication of resection for vault failure after hysterectomy and radiation included the following: 1) superficially carcinoma, 2) an interval of greater than 6 months between the initial operation and vault failure, and 3) consensus for the presence of both operative and postoperative complications. In this study, we have reported eight patients with vault failures who underwent vault resection.

Choi *et al*. reported four cases of laparoscopic upper vaginectomy for vaginal intraepithelial neoplasia and superficially invasive vaginal carcinoma after hysterectomy without radiotherapy [6]. Their operations were adapted for a recurrent lesion in the upper third of the vagina, and laparoscopic upper vaginectomy succeeded in controlling these cases without second vault failure.

Panici *et al*. reported cases in which vaginectomies were performed in patients who underwent radical hysterectomy without pelvic radiotherapy [7]. In this series, early postoperative complications were experienced by seven of the patients who had undergone vaginal vaginectomy: four patients developed vaginal scar abscess dehiscence, two experienced postoperative bleeding, and one developed a vesicovaginal fistula. Six subsequent complications were also observed: three patients developed vaginal stenosis, two patients developed urinary incontinence, and one patient developed ureteral stenosis. After vaginectomy, seven patients developed metastases, but no patient developed a second vaginal failure. Five-year overall and progression-free survival rates were 70.5% and 59.4%, respectively. Panici *et al*. concluded that vaginal vaginectomy was feasible in selected patients. Their study’s selection criteria were as follows: patients were included if they had vaginal lesions <2 cm in size that were not juxtaposed to the rectum or bladder, and patients were excluded if they had prior radiation, prior nodal metastasis, palpable paravaginal invasion, or positive results in examinations for metastasis (including positron emission tomography (PET)). The criteria for performing a pre-operative PET-computed tomography were the absence of both bulky tumor and distant disease.

In contrast to these previous reports, radiotherapy had been already performed in our study because of its good efficacy. At the second vault failure after radiotherapy, resection of the vault was performed via laparotomy. Total vaginectomy resulted in complications (grade 3, 37.5%; 3/8) because of the adherence of the bladder and rectum after the first surgery and radiotherapy. Bladder filling was used to identify the altered anatomy after prior hysterectomy, as well as the induration of the vaginal vault leading to the bladder, vaginal apex, and border. Postoperative repeated cystitis and neurogenic bladder required longer hospitalization. Three patients developed vesicovaginal fistulae, which are associated with a high risk of severe complications. Vesicovaginal fistulae were managed conservatively, and fortunately, each of the patient’s fistulae closed spontaneously. Technically feasible procedures could result in maintained bladder or bowel function.

## Conclusions

Although this report is somewhat limited by the small number of patients, our results suggest that vaginectomy after radiotherapy is effective. In this series, local control was achieved in five cases. Since vaginal resection can be performed to achieve local control, this surgery is an option for vaginal vault recurrence, as is pelvic exenteration. However, an earlier study showed higher complication rates when this operation was performed after hysterectomy and brachytherapy. Because of the associated complications, vaginectomy should only be selected after carefully considering possible alternatives. For patients who have been selected carefully, vaginal resection is an acceptable treatment option.

Because of the rarity of the cases that we have considered, single-institution studies with large sample are likely to be infeasible. We suggest that data pooling is the most promising avenue for identifying optimal therapies and achieving other progress in this field. Such data pooling could come in the form of a review of all previously published case reports, case series, and small studies; the organization of prospective, multicenter case series; and/or the referral of all similar cases in Japan to a select group of medical institutes.

### Consent

Written informed consent was obtained from the patient for the publication of this report and any accompanying images.

## References

[1] International Agency for Research on Cancer. Cervical cancer estimated incidence, mortality and prevalence worldwide in 2012. World Health Organization; 2012. Available at: http://globocan.iarc.fr/Pages/fact_sheets_cancer.aspx?cancer=cervix

[2] Yamagami W, Aoki D (2014). Annual report of the committee on gynecologic oncology, the Japan Society of Obstetrics and Gynecology. J Obstet Gynaecol Res.

[3] Thomas GM, Dembo AJ, Myhr T, Black B, Pringle JF, Rawlings G (1993). Long-term results of concurrent radiation and chemotherapy for carcinoma of the cervix recurrent after surgery. Int J Gynecol Cancer.

[4] Jobsen JJ, Leer JW, Cleton FJ, Hermans J (1989). Treatment of locoregional recurrence of carcinoma of the cervix by radiotherapy after primary surgery. Gynecol Oncol.

[5] Ito H, Shigematsu N, Kawada T, Kubo A, Isobe K, Hara R (1997). Radiotherapy for centrally recurrent cervical cancer of the vaginal stump following hysterectomy. Gynecol Oncol.

[6] Choi YJ, Hur SY, Park JS, Lee KH (2013). Laparoscopic upper vaginectomy for post-hysterectomy high risk vaginal intraepithelial neoplasia and superficially invasive vaginal carcinoma. J Surg Oncol.

[7] Panici PB, Manci N, Bellati F, Di Donato V, Marchetti C, De Falco C (2009). Vaginectomy: a minimally invasive treatment for cervical cancer vaginal recurrence. Int J Gynecol Cancer.

[8] Nakayama K, Teshima H, Hirai Y, Hasumi K, Masubuchi K (1990). Stump recurrence after radical hysterectomy for patients with uterine cervical cancer (in Japanese). Nihon Sanka Fujinka Gakkai Zasshi.

[9] Berek JS, Howe C, Lagasse LD, Hacker NF (2005). Pelvic exenteration for recurrent gynecologic malignancy: survival and morbidity analysis of the 45-year experience at UCLA. Gynecol Oncol.

[10] Ferenschild FTJ, Vermaas M, Vehoef C, Ansink AC, Kirkels WJ, Eggermont AMM (2009). Total pelvic exenteration for primary and recurrent malignancies. World J Surg.

